# Alteration of renal respiratory Complex-III during experimental type-1 diabetes

**DOI:** 10.1186/1472-6823-9-2

**Published:** 2009-01-23

**Authors:** Shankar Munusamy, Hamida Saba, Tanecia Mitchell, Judit K Megyesi, Robert W Brock, Lee Ann MacMillan-Crow

**Affiliations:** 1 Department of Pharmacology & Toxicology, Division of Nephrology, College of Medicine, University of Arkansas for Medical Sciences, Little Rock, AR 72205, USA; 2 Department of Internal Medicine, Division of Nephrology, College of Medicine, University of Arkansas for Medical Sciences, Little Rock, AR 72205, USA; 3 Department of Physiology & Pharmacology and Center for Interdisciplinary Research in Cardiovascular Sciences, West Virginia University School of Medicine Morgantown, WV 26506, USA

## Abstract

**Background:**

Diabetes has become the single most common cause for end-stage renal disease in the United States. It has been established that mitochondrial damage occurs during diabetes; however, little is known about what initiates mitochondrial injury and oxidant production during the early stages of diabetes. Inactivation of mitochondrial respiratory complexes or alteration of their critical subunits can lead to generation of mitochondrial oxidants, mitochondrial damage, and organ injury. Thus, one goal of this study was to determine the status of mitochondrial respiratory complexes in the rat kidney during the early stages of diabetes (5-weeks post streptozotocin injection).

**Methods:**

Mitochondrial complex activity assays, blue native gel electrophoresis (BN-PAGE), Complex III immunoprecipitation, and an ATP assay were performed to examine the effects of diabetes on the status of respiratory complexes and energy levels in renal mitochondria. Creatinine clearance and urine albumin excretion were measured to assess the status of renal function in our model.

**Results:**

Interestingly, of all four respiratory complexes only cytochrome c reductase (Complex-III) activity was significantly decreased, whereas two Complex III subunits, Core 2 protein and Rieske protein, were up regulated in the diabetic renal mitochondria. The BN-PAGE data suggested that Complex III failed to assemble correctly, which could also explain the compensatory upregulation of specific Complex III subunits. In addition, the renal F_0_F_1_-ATPase activity and ATP levels were increased during diabetes.

**Conclusion:**

In summary, these findings show for the first time that early (and selective) inactivation of Complex-III may contribute to the mitochondrial oxidant production which occurs in the early stages of diabetes.

## Background

Renal dysfunction is a major complication that affects 20–40% of individuals with diabetes [1] and diabetic nephropathy is the most common cause of end stage renal disease (ESRD) in the United States [2]. Currently, no treatment options are available to prevent the renal complications of diabetes except for therapies which may slow the progression through intensive control of blood glucose and blood pressure [3]. Thus, it is important to identify new therapeutic targets that might lead to the prevention of diabetes-induced nephropathy.

Several reports indicate that hyperglycemia-induced generation of superoxide within the mitochondria plays a major role in the development of diabetic complications [4-7]. Nevertheless, the role that hyperglycemia has on renal mitochondrial respiratory complex function has not been thoroughly investigated. A few studies have indicated that diabetes induces alterations in the activities of mitochondrial respiratory complexes and mitochondrial respiration in the kidney [8,9]. Rosca et al. demonstrated that renal Complex-III was a target for glycation and inhibition during chronic diabetes (12 months) [8]. A study by Katyare and Satav showed that respiration rates and ATPase activity were elevated in diabetic renal mitochondria. This study also demonstrated that renal mitochondria were tightly coupled during diabetes [9]. In addition, Complex-III has also been shown to be inhibited during chronic diabetes in diabetic mouse retina [10]. Among the four respiratory complexes I through IV that comprise the electron transport chain, Complex-I and III are thought to be the major sources for superoxide generation within the mitochondria [11]. However, the effect that short-term hyperglycemia has on renal mitochondrial complexes has not been examined in detail, and may lead to a more precise understanding of the initiating events involved with mitochondrial oxidant production during diabetes.

In this study, we have examined the status of renal mitochondrial complexes during the early stages (5-weeks) of experimental Type-1 diabetes using the streptozotocin-induced diabetes rat model. Our findings provide evidence for the first time that short-term diabetes caused selective Complex-III inactivation (with no effect on Complex I, II, or IV). In addition, further studies indicated that two Complex III subunits were induced following diabetes. These results suggest that in the early stages of hyperglycemia Complex III has assembly defects which could cause oxidant generation, leading to impairment of mitochondrial and renal function during hyperglycemia.

## Methods

### Streptozotocin-induced diabetes model

Male Sprague Dawley rats weighing 200 ± 50 g obtained from Harlan Sprague Dawley Inc. were used for this study. All of the animal protocols were approved by the Institutional Animal Care and Use Committee at the University of Arkansas for Medical Sciences to perform as described in the paper. Rats were randomly divided into two groups: control and diabetic. Diabetes was induced by injecting overnight fasted rats with streptozotocin (STZ; 65 mg/kg, i.p.) dissolved in 0.5 ml saline, while the control rats were injected with 0.5 ml saline. Fasting blood glucose levels were measured 48 hours post-STZ injection and rats with blood glucose levels greater than 250 mg/dl were classified as "diabetic". Diabetes-induced changes were studied at 5 weeks post-STZ injection (n = 6).

### Metabolic studies, organ harvest and blood collection

Animals were kept in metabolic cages and acclimatized overnight prior to urine collection. Urine was collected from each rat for 24 hr, and their 24 hr food and water intake were also measured. Animals were sacrificed under isoflurane anesthesia and their kidneys harvested. Blood was collected via intracardiac puncture and serum samples were stored at -80°C until used for creatinine measurement.

### Creatinine clearance & urine albumin measurement

Serum creatinine (sCr) and urine creatinine (uCr) levels were measured by a modified Jaffe's method (Pointe Scientific, Canton, MI, USA) in a Cobas Mira clinical analyzer (Roche Diagnostics, Indianapolis, IN, USA). Creatinine clearance (CrCl) was then calculated from the formula: CrCl (ml/min/kg) = (uCr × uVolume × Body weight)/(sCr × 1440 min × 1000). Urine albumin levels were determined by an immunoturbidometric assay (Pointe Scientific, Canton, MI, USA) in a Cobas Mira clinical analyzer and expressed as mg/day/kg.

### Histological scoring

Periodic acid-Schiff (PAS) staining was performed on formalin fixed, paraffin embedded kidney sections to assess tubular integrity and renal morphology as previously described [12,13]. Tissue sections were scored on a 0 to 4 scale by two pathologists blinded to the treatment groups according to the extent of tubular dilation and glomerular changes as follows: 0 = absent; 1 = mild; 2 = moderate; 3 = severe; 4 = very severe.

### Measurement of respiratory complex activities

Renal mitochondria were isolated from whole kidneys by differential centrifugation in a sucrose-containing buffer as previously described [14,15]. The activity of mitochondrial Complexes-I through IV as described by Birch-Machin et al. [16] with minor modifications as previously described [15].

**F_o_F_1_-ATP-ase (Complex-V) activity **was assayed by measuring the release of P_i _from ATP by the method of Law et al.[17]. Each sample was run in the presence and absence of oligomycin (10 μg/ml), and the F_o_F_1_-ATP-ase activity was expressed as the oligomycin-sensitive ATPase activity.

### Blue Native Gel Electrophoresis (BN-PAGE)

Mitochondrial complexes were analyzed by performing BN-PAGE on the control and diabetic mitochondria as previously described [15]. To confirm the identity of mitochondrial Complex-III following BN-PAGE, the native gel was directly transferred to a PVDF membrane after first dimension and probed for known Complex III subunits Core 2 and Rieske subunits as described below. In addition, immunoblot analysis for Rieske and Core 2 subunits was performed following two-dimensional BN-PAGE by performing BN-PAGE, excising the Complex III bands, followed by SDS-PAGE in the second dimension.

### ATP Assay

ATP levels were determined in the mitochondrial fractions of control and diabetic rat kidneys using a luciferase based bioluminescent assay kit (Sigma-Aldrich, St. Louis, MO, USA) in a TD 20/20 luminometer (Turner Designs, Sunnyvale, CA, USA).

### Immunoprecipitation of Complex-III

Complex-III was immunoprecipitated from control and diabetic rat renal mitochondria (2.5 mg) as described by Shilling et al. [18] using Complex-III immunocapture beads (MS301) from Mitosciences, USA. After SDS-PAGE, the gels were stained with Coomassie blue staining solution (0.1% bromophenol blue R-250 in 40% methanol and 10% acetic acid) for 30 minutes and then destained overnight in 7.5% acetic acid/5% methanol solution.

For immunoblotting, the gel was transferred to a PVDF membrane, and probed for Core 2 protein or the Rieske subunit using mouse monoclonal antibodies – MS304 or MS305 (Mitosciences) at 1:1,000 dilution overnight at 4°C. Blots were then probed with peroxidase labeled goat anti-mouse secondary antibody, and developed using an enhanced chemiluminescent substrate system.

### Statistical Analysis

Data analysis was performed using Origin 6.0. All values were expressed as mean ± SEM. Independent t-test (two-tailed) was used to compare the mean values between control and diabetic groups. Differences were considered statistically significant if the p values were less than 0.05.

## Results

### Diabetic Rat Model

Rats made diabetic with STZ had fasting blood glucose levels of ~350 mg/dl, 48 hours after injection. Consistent with numerous reports, our diabetic rats (5-weeks post-STZ) showed a significant elevation in fasting blood glucose, 480.0 ± 69.3 mg/dl Vs 66.3 ± 3.8 mg/dl in an age-matched control group.

Diabetic rats also exhibited the typical hallmark features of diabetes: polyuria, polydipsia, polyphagia, and a significant decrease in body weight compared to the control group (Table 1).

**Table 1:** Metabolic effects of STZ-induced diabetes in rats.

**Parameters**	**Control**	**Diabetic**
Fasting blood glucose (mg/dl)	66.3 ± 3.8	480.0 ± 69.3*

Body weight (BW) (g)	371 ± 9.5	260.6 ± 17.6*

Food Intake (g/100 g BW)	5.7 ± 0.3	14.3 ± 0.4*

Water Intake (ml/100 g BW)	9.4 ± 1.7	73.2 ± 3.1*

Urine Output (ml/100 g BW)	3.4 ± 0.3	52.1 ± 2.9*

This table shows the effect of STZ-induced diabetes (5-week post-STZ) on fasting blood glucose, food intake, water intake, and urine output in male Sprague-Dawley rats. All values are expressed as Mean ± SEM (n = 6). * P < 0.05 compared with age-matched (5-week) controls.

### Diabetes induces hyperfiltration, albuminuria, and renal damage

**Figure 1:**
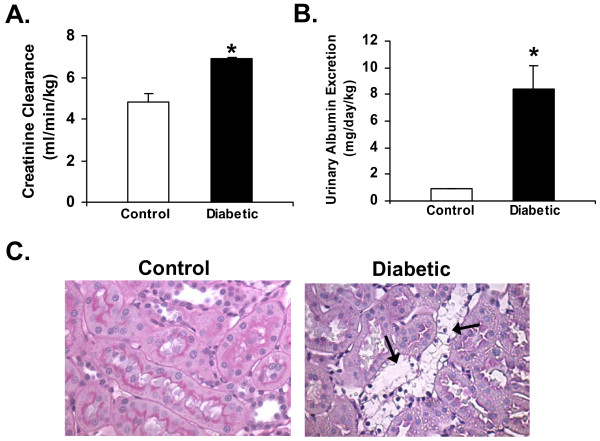
**Diabetes-induced renal dysfunction**. Effect of STZ-induced diabetes (5-week post-STZ) on, **A) **Creatinine clearance, and **B) **Urinary albumin excretion in male Sprague-Dawley rats. Values are expressed as Mean ± SEM (n = 6). * P < 0.05 compared with age-matched (5-week) controls. **C) **Periodic acid-Schiff (PAS) stained sections of control and (5-week post-STZ) diabetic rat kidneys observed under 400× magnification. Arrows indicate regions of renal damage. Experiments were repeated three times with similar results.

Creatinine clearance, a measure of glomerular filtration rate, was used to monitor kidney function, and urinary albumin excretion was used as a marker for early development of renal injury in our diabetic rat model. A significant elevation in creatinine clearance was observed in the diabetic group (6.53 ± 0.36 ml/min/kg) compared to the age-matched control group (4.80 ± 0.42 ml/min/kg), indicating STZ-induced diabetes caused hyperfiltration in the kidney (Figure 1A). In addition, STZ-injected rats also showed a significant increase in urinary albumin excretion (8.43 ± 1.74 mg/day/kg in diabetic group Vs 0.9 ± 0.03 mg/day/kg in age-matched controls) (Figure 1B).

Periodic Acid-Schiff (PAS) staining (which stains carbohydrates in brush borders, tubules, and glomerular basement membrane) was used to assess renal damage in the diabetic rat kidneys. Histological scoring of PAS stained kidney sections by two pathologists revealed that diabetes caused a moderate renal damage (a score of 0.17 in control Vs 2.0 in diabetic group) (Figure 1C).

### Hyperglycemia induces specific alterations to renal mitochondrial respiratory complexes

To examine whether early diabetes had a significant impact on the renal mitochondrial respiratory chain, we used a relatively novel technique called Blue Native-Polyacrylamide Gel Electrophoresis (BN-PAGE). BN-PAGE directly detects the membrane bound mitochondrial respiratory complexes and, thus, can pinpoint which of the five respiratory complexes (I-V) show the most intense change in levels and assembly during diabetes [19].

**Figure 2:**
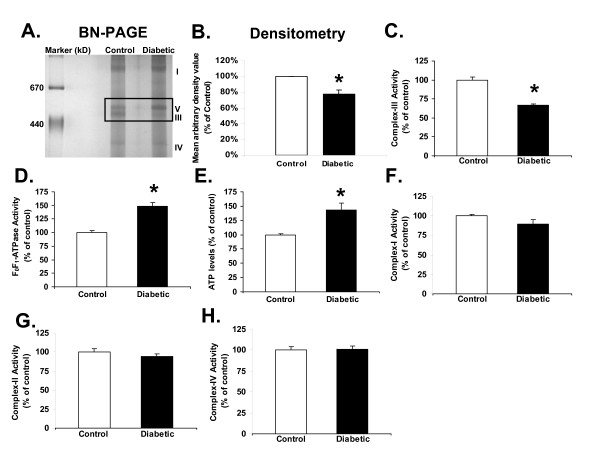
**Diabetes-induced alterations in the activity of mitochondrial complexes and ATP levels**. **A) **Blue Native Gel Electrophoresis (BN-PAGE) showing alterations in mitochondrial complex proteins during diabetes. The box represents Complexes-III and V that differ in control and diabetic (5-week post-STZ) mitochondria. **B) **Densitometric analysis of BN-PAGE showing a decrease in Complex III levels during diabetes. **(C-H) **Bar graphs indicating inactivation of mitochondrial Complex-III (**C)**, and induction of F_0_, F_1_-ATPase (Complex-V) activity (**D)**, and ATP levels **(E) **in diabetic rat kidney as compared to controls. Activities of renal mitochondrial complexes: **F) **Complex-I, **G) **Complex-II, and **H) **Complex-IV, were not altered by diabetes. All values are expressed as percentage Mean ± SEM (n = 5) of controls (set to 100). * P < 0.05 compared with age-matched controls.

The BN-PAGE data indicated that Complex-III was decreased (77.8% of control) and Complex-V was increased in the diabetic kidneys compared to the control group (Figure 2A and 2B). In addition to BN-PAGE, respiratory complex activity assays were performed which further confirmed that Complex-III activity was significantly reduced in diabetic renal mitochondria (66.32 ± 1.93%) as compared to the age-matched controls (100 ± 3.58%, Figure 2C); while F_0_F_1_-ATPase (Complex-V) activity was increased (148.71 ± 6.45% in diabetic Vs 100 ± 3.37% in control; Figure 2D). Consistent with increased F_0_F_1_-ATPase activity, ATP levels were also significantly increased in diabetic rat kidneys (143.39 ± 11.56%) compared to the age-matched controls (100 ± 2.01%; Figure 2E). Interestingly, no significant changes were observed in the activities of Complexes I, II and IV between the diabetic and control groups (Figure 2F–H). It is important to realize that Complex III contains 11 subunits. Hence, further studies were performed to identify the specific subunits within Complex-III that might be altered during hyperglycemia using Complex III immunoprecipitation.

**Figure 3:**
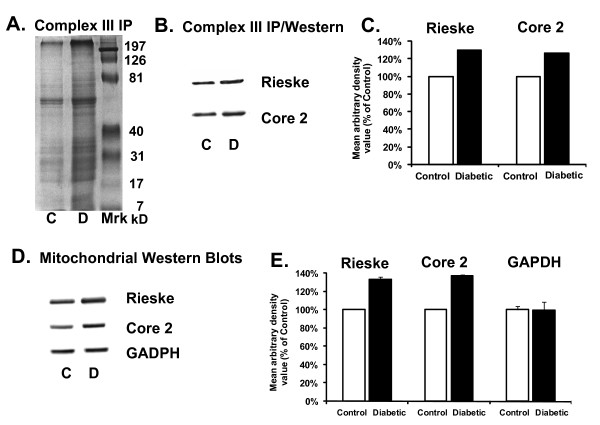
**Diabetes-induced upregulation of renal Complex-III subunits, Rieske protein and Core 2 protein**. **A) **Coomassie blue stained SDS-Polyacrylamide gel (following immunoprecipitation of Complex-III in control (C) and diabetic (D) rat kidney mitochondria) revealing upregulation of mitochondrial Complex-III subunit proteins during diabetes. **B) **Complex III IP followed by Western blot analysis revealed upregulation of Complex III associated Rieske Protein and Core 2 Protein subunits during diabetes. **C) **Densitometric analysis showing Complex III associated subunits were upregulated during diabetes. **D) **Rieske Protein and Core 2 Protein Western blot analysis of total mitochondria. GAPDH Western blot analysis was used as a loading control. **E) **Densitometric analysis of the Western blot shown in panel D. Blots are representative of three separate experiments.

Surprisingly, Complex III immunoprecipitation in the diabetic samples yielded more proteins associated with Complex III when compared to control samples as visualized by Coomassie staining (Figure 3A). The precise reason for the enhanced protein staining in the diabetic mitochondria remains unclear, but may be related to altered assembly of Complex III. Nevertheless, we chose to focus on the key redox sensitive subunit within Complex III, the Rieske protein as well as the Core 2 protein, a subunit that is not directly involved with electron transfer.

Thus, Western blot analysis was performed for Rieske or Core 2 protein following Complex III immunoprecipitation. Diabetes appeared to increase Complex III associated Rieske and Core 2 proteins when compared to control kidneys (Figure 3B and 3C). The increased expression of specific Complex III subunits was further confirmed by Western blot analysis using renal mitochondria (without immunoprecipitation) which also showed an upregulation of the Complex-III subunits, Rieske (133.33 ± 3.1%) and Core 2 protein (136.69 ± 1.44%) during diabetes (Figure 3D and 3E) as compared to control. GAPDH was used as the loading control.

**Figure 4:**
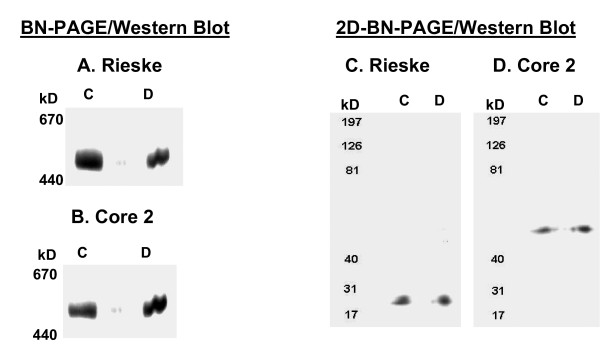
**Identification of renal Complex-III following BN-PAGE**. Western blot analysis for Rieske Protein **(A and C) **and Core 2 Protein **(B and D) **to confirm the identity of the Complex-III band after one dimension BN-PAGE (left panel/A and B) and 2D-BN-PAGE (right panel/C and D) using control (C) and diabetic (D) rat kidney mitochondria.

Additional experiments were carried out to verify the identity of Complex III following BN-PAGE. These included Western blotting for Rieske and Core 2 proteins after BN-PAGE (Figure 4A &4B) as well as BN-PAGE followed by SDS-PAGE in the second dimension (2D BN-PAGE) (Figure 4C &4D). These data clearly demonstrated that the band identified as Complex III in the BN-PAGE contains both the Rieske and Core 2 subunits of Complex III.

## Discussion

These results indicate that renal Complex-III appears to be an early and specific mitochondrial target during experimental type-1 diabetes. Complex-III is centrally located in the electron transfer process and has been implicated as one of the major sites for superoxide generation in the mitochondria during diabetes [8,10]. Partial inhibition of Complex-III during conditions of increased respiration [9] would decrease the transfer of electrons from ubiquinol to Complex-III, and increase the half-life of ubisemiquinone, which leads to generation of superoxide [8,10].

Intriguingly, our results indicated an upregulation of renal Complex-III subunits, Core 2 and Rieske protein during diabetes. However, using BN-PAGE it was shown that Complex III was not correctly assembled (decreased levels on BN-PAGE) which also correlated with a decrease in Complex-III activity. Thus, the paradoxical increase in expression of individual subunits, Core 2 and Rieske proteins could be a compensatory response to restore the activity of Complex-III during diabetes.

Rieske subunit is a nuclear encoded protein that is imported into the mitochondrial matrix where it undergoes a two-step cleavage of its pre-sequence and is translocated into the inner mitochondrial membrane. It has an iron-sulfur [2Fe-2S] redox center that centrally participates in the Q cycle by transferring an electron from ubiquinol to cytochrome c_1_, which then donates it to cytochrome c. Core 2 subunit, which is also a nuclear encoded protein is a homolog of mitochondrial processing protease-alpha subunit and is thought to be important for mitochondrial protein import, processing and integrity of Complex-III [20]. Although, Core 2 subunit is not primarily involved in electron transfer, genetic deletion of Core 2 has been shown to affect the assembly of Complex-III [21]. Thus, an increase in the Rieske as well as the Core 2 subunits suggests a possible assembly defect in Complex-III.

To our knowledge, this is the first report demonstrating that renal Complex-III and its subunits are altered in the early stages of diabetes. A study by Rosca et al. showed previously that Complex-III was a target for glycation by methyl glyoxal and inactivated during chronic diabetes (12 months) [8], while no significant changes were reported at 2 months of diabetes. Although, the reason for this discrepancy remains unknown; one difference could be the fraction of the kidney analyzed in the two studies. Our study utilized whole rat kidneys, whereas the study by Rosca et al. used the cortical fractions of kidneys. Although the renal cortex has been primarily considered to be the energetically active segment of the nephron, several studies suggest that the medullary thick ascending limb (mTAL) is an active site for reabsorption in kidney and thus might play a major role in mitochondrial superoxide production. In addition, increased oxidant production has been observed in both the cortical and medullar regions of the kidney. Thus, we felt it was important to include both cortex and medulla (whole kidney) for mitochondrial isolation and study the net effect of diabetes on the renal mitochondria.

Despite the findings by Katyare and Satav [9] who showed increased cytochrome aa3 contents in renal mitochondria during diabetes, we did not detect any significant changes in Complex IV activity in our rat model of diabetes. It is also important to note that the authors could not correlate the changes in cytochrome content with the altered respiration rates observed during diabetes. In addition, these authors did not measure Complex IV activity and we did not measure cytochrome aa3 contents so a direct comparison of these studies is difficult to assess. Again, one important difference between the two studies is the source of renal mitochondria used: we used whole kidney mitochondria, while Katyare and Satav used cortical mitochondria as discussed above.

Our results also showed that F_0_F_1_-ATPase activity and renal ATP levels were significantly increased in the diabetic renal mitochondria compared to controls. Interestingly, the study by Katyare and Satav [9] also demonstrated that respiration and ATPase activity were increased in diabetic renal mitochondria. Thus, it is possible that the increased respiration leads to increased proton motive force (hyperpolarization) and increased ATP levels which serve to fulfill the increased energy demands of the kidney during diabetes. Concomitantly, the increased respiration would likely result in more superoxide (oxidant) generation which could lead to alterations in Complex III. In fact, we have observed that renal proximal tubule cells exposed to hyperglycemia (25 mM; 48 hr) results in mitochondrial hyperpolarization, Complex-III inhibition, and oxidant production (manuscript under review).

## Conclusion

In conclusion, we have demonstrated that early stages of diabetes induced alterations in Complex-III, increased ATP synthase activity, and renal dysfunction. These diabetes-induced alterations in activities of mitochondrial complexes and energy status could contribute to the underlying role of oxidative stress in the pathogenesis of diabetic nephropathy. Our future *in vitro *studies will explore the mechanistic pathways by which hyperglycemia induces mitochondrial complex dysfunction and oxidant production. Finally, strategies to limit the extent of renal mitochondrial damage during hyperglycemia (by therapeutic agents that will specifically modulate mitochondrial function) might prevent or inhibit the development of nephropathy in the diabetic population.

## Abbreviations

BN-PAGE: Blue Native Polyacrylamide Gel Electrophoresis; Complex-III: Ubiquinol:Cytochrome c oxidoreductase or Cytochrome bc1 complex; CrCl: Creatinine Clearance; ESRD: End Stage Renal Disease; GAPDH: Glyceraldehyde-3-Phosphate Dehydrogenase; PAS: Periodic acid-Schiff; sCr: Serum Creatinine; STZ: Streptozotocin; uCr: Urine Creatinine.

## Competing interests

The authors declare that they have no competing interests.

## Authors' contributions

SM and LM were responsible for the overall design of the study. SM performed most of the biochemical analyses and renal function studies. TM and HS provided technical guidance and advice for the biochemical methods. JM performed the histological assessment. JM, RB and LM critically reviewed the manuscript, and played an important role with regard to intellectual content. SM wrote the draft of the manuscript. All the authors have read and approved the final manuscript.

## Pre-publication history

The pre-publication history for this paper can be accessed here:


